# A compassionate imagery intervention for patients with persecutory delusions

**DOI:** 10.1017/S1352465821000229

**Published:** 2022-01

**Authors:** Ava Forkert, Poppy Brown, Daniel Freeman, Felicity Waite

**Affiliations:** 1 Oxford Institute of Clinical Psychology Training, Oxford, UK; 2 Department of Psychiatry, University of Oxford, Oxford, UK; 3 Oxford Health NHS Foundation Trust, Oxford, UK

**Keywords:** delusions, intervention, psychosis, schizophrenia, self-esteem, therapy

## Abstract

**Background:**

Negative beliefs about the self, including low self-compassion, have been identified as a putative causal factor in the occurrence of paranoia. Therefore, improving self-compassion may be one route to reduce paranoia.

**Aims:**

To assess the feasibility, acceptability, and potential clinical effects of a brief compassionate imagery intervention for patients with persecutory delusions.

**Method:**

Twelve patients with persecutory delusions received an individual four-session compassionate imagery intervention. Assessments of self-concept and paranoia were completed before treatment, immediately after treatment, and at 1-month follow-up. A qualitative study exploring participants’ experiences of the treatment was also completed.

**Results:**

Twelve out of 14 eligible patients referred to the study agreed to take part. All participants completed all therapy sessions and assessments. Post-treatment, there were improvements in self-compassion (change score –0.64, 95% CI –1.04, –0.24, *d* = –1.78), negative beliefs about the self (change score 2.42, 95% CI –0.37, 5.20, *d* = 0.51), and paranoia (change score 10.08, 95% CI 3.47, 16.69, *d* = 0.61). There were no serious adverse events. Three themes emerged from the qualitative analysis: ‘effortful learning’, ‘seeing change’ and ‘taking it forward’. Participants described a process of active and effortful engagement in therapy which was rewarded with positive changes, including feeling calmer, gaining clarity, and developing acceptance.

**Conclusion:**

This uncontrolled feasibility study indicates that a brief compassionate imagery intervention for patients with persecutory delusions is feasible, acceptable, and may lead to clinical benefits.

## Introduction

Persecutory delusions are unfounded beliefs that others intend harm to the person. In our cognitive understanding of paranoia, these are threat beliefs that build upon a sense of vulnerability partly arising from negative beliefs about the self as different, inferior and apart (Freeman, 2016). An extensive literature reports that paranoia is associated with negative cognitions about the self (e.g. Kesting and Lincoln, 2013; Tiernan *et al*., 2014). Consistent with a causal role, experimental studies have found that altering negative beliefs about the self alters levels of paranoia (e.g. Freeman *et al*., 2014; Atherton *et al*., 2016). Negative self-beliefs are pervasive in patients with paranoia (Collett *et al*., 2016; Freeman, 2007; Garety and Freeman, 1999). For example, a recent survey of 1800 patients with non-affective psychosis in NHS mental health services showed that a third held strongly negative views of the self (Freeman *et al*., 2019). Importantly, this study highlighted that patients want treatment to increase their confidence. In patients with persecutory delusions, negative self-beliefs and low levels of self-compassion are highly associated (Collett *et al*., 2016). Hence one potential treatment method through which negative self-beliefs could be improved is by increasing self-compassion.

Self-compassion is defined as kindness towards oneself, which incorporates an awareness of our own suffering, understanding our experience of suffering as part of our common humanity (Neff, 2003a,b), and a motivation to alleviate suffering (Gilbert, 2010a,b). In compassion focused therapy (CFT; Gilbert, 2009; Gilbert, 2010a,b), imagery techniques are used to enhance levels of compassion and regulate feelings of threat. The effects of compassionate imagery techniques on paranoia have been tested in experimental manipulation studies. In students with unselected levels of paranoia, compassionate imagery techniques led to improvements in self-compassion, self-esteem and paranoia (Lincoln *et al*., 2013). In people from the general population reporting high levels of paranoia, compassionate imagery led to large effect size improvements in self-compassion and paranoia (Brown *et al*., 2020). A mediation analysis indicated that change in self-compassion explained 57% of the change in paranoia. Yet in one study it was found that a single session of compassionate imagery did not result in improvements in paranoia in patients with a history of persecutory delusions (Ascone *et al*., 2017). This was presumably a consequence of the brief intervention of Ascone *et al*. (2017) not increasing levels of the hypothesised mediator, self-compassion. As self-compassion did not increase with this intervention, a reduction in paranoia would not be expected. Moreover, given the severity, and often chronicity, of persecutory delusions it is likely that a longer intervention is required. Research on the use of imagery in psychological interventions has shown that imagery is a powerful tool in changing people’s emotional states but that rehearsal is important for effectiveness (Hackmann *et al*., 2011).

The current study investigates a four-session compassionate imagery intervention for patients with persecutory delusions. The primary aim of the study was to assess the acceptability and feasibility of the intervention. The secondary aim was to provide an initial indication of potential clinical benefits. Feasibility and acceptability were assessed by recruitment rate, data completion, uptake of the intervention, monitoring of adverse events or adverse reactions, as well as quantitative and qualitative feedback from participants. We sought an initial signal of clinical efficacy by measuring change in self-compassion, negative self-beliefs, and paranoia, immediately post-treatment and at 1-month follow-up.

## Method

### Design

This was an uncontrolled feasibility study. Assessments were conducted at baseline (0 weeks), post-treatment (2 weeks), and at 1-month follow-up (6 weeks). Qualitative interviews were conducted by an independent researcher (P.B.) at the 1-month follow-up appointment. To increase methodological quality and reporting of the qualitative interviews, the 32-item consolidated criteria for reporting qualitative research (COREQ; Tong *et al*., 2007) were followed. All other assessments were conducted by the study therapist (A.F.). The study received approval from an NHS research ethics committee (reference: 18/LO/1555).

### Service user involvement

The design and name of the study, and all patient-facing documents including therapy materials, were created in collaboration with people with lived experience of psychosis through the Oxford Cognitive Approaches to Psychosis Patient Advisory Group facilitated by the McPin Foundation.

### Participants

Twelve participants were recruited from secondary mental health services in the Oxford Health NHS Foundation Trust and Berkshire Healthcare NHS Foundation Trust. Written informed consent was obtained prior to participation. The inclusion criteria were: patients aged 18–65 years; with a persistent persecutory delusion (duration at least 3 months); a clinical diagnosis of non-affective psychosis; a minimum score of 29 on the Green *et al*. Paranoid Thoughts Scales (GPTS) Part B persecutory ideation sub-scale (Green *et al*., 2008) at screening, consistent with the cut-off for a randomised controlled trial for persecutory delusions (Garety *et al*., 2017); and stable psychiatric medication for at least 1 month. The exclusion criteria were: current receipt of individual psychological therapy for psychosis; insufficient comprehension of English to complete the therapy and assessments; a primary diagnosis of alcohol or drug addiction, organic syndrome or personality disorder; or a moderate-to-severe learning disability.

### Markers of acceptability and feasibility

Feasibility and acceptability were assessed by recruitment rate (percentage uptake by suitable participants), data completion, uptake of the intervention (attendance and completion), treatment adherence, monitoring of adverse events, as well as quantitative and qualitative feedback from participants.

#### Participant feedback

At the post-treatment assessment, participants were given a therapy feedback questionnaire. This questionnaire was designed specifically for this study. It was informed by the Patient Experience Questionnaire used in local NHS services and was adapted in consultation with people with lived experience of psychosis. At the 1-month follow-up, participants were invited to take part in a semi-structured interview (see Supplementary material: Measure 1) to gather qualitative data about their experiences of participating in the study.

#### Adverse events

Serious adverse events were defined prior to the study as: medical occurrences resulting in death, life-threatening illness, leading to or prolonging hospitalisation or resulting in significant disability. In addition to patient report, medical records were screened to identify any adverse events.

### Measures of clinical outcomes

To provide an initial assessment of potential clinical benefits, participants were given the same core battery of assessments at all three time points. Demographic information was collected at the baseline assessment.

#### Green et al. Paranoid Thoughts Scales (GPTS; Green et al., 2008)

This self-report questionnaire is a 16-item measure of ideas of reference and persecution. The GPTS Part A assesses ideas of social reference, such as ‘I often hear people referring to me’, while the GPTS Part B assesses persecutory ideation, such as ‘people have intended me harm’. Each item is scored on a Likert scale of 1 (not at all) to 5 (totally). The total scores on each sub-scale range from 16 to 80. The higher the score, the higher the level of paranoia. For the GPTS Part B, suggested score categories include: 16–23 (average), 24–34 (elevated), 35–44 (moderately severe), 45–59 (severe), 60+ (very severe), with a cut-off for persecutory delusions of 45 (Freeman *et al*., 2019). The GPTS has high internal consistency (α = .90 for both sub-scales) and good levels of internal reliability (*r =* .88 for GPTS Part A and *r =* .81 for GPTS Part B; Green *et al*., 2008). The questionnaire uses a time frame of 2 weeks, which was used at baseline, so that the cut-off for inclusion in this study is comparable to the inclusion criteria for other studies in this clinical population. Due to the short time frame of the intervention, the time frame was adapted to 1 week at post-treatment and follow-up in order to be sensitive to change.

#### Self-Compassion Scale (Neff, 2003b)

The Self-Compassion Scale is a 26-item self-report measure and the most widely used measure of self-compassion. Participants rate each statement (e.g. ‘I’m tolerant of my own flaws and inadequacies’) on a Likert scale of 1 (almost never) to 5 (almost always), some of which are reverse scored. Higher total scores indicate greater self-compassion when distressed. Internal consistency has been found to be high (α = .92), as has the test–re-test reliability (*r = .*93; Neff, 2003b). A 1-week time frame was used.

#### Brief Core Schema Scale (BCSS; Fowler et al., 2006)

This 24-item self-report measure assesses both positive and negative beliefs about the self and others over the last week on a scale of 0 (do not believe it) to 4 (believe it totally). Scores are summed for four sub-scales: negative self, positive self, negative others and positive others. The higher the score on the positive sub-scales, the more positive the view about the self or others. In contrast, the higher the score on the negative sub-scales, the more negative the view about the self or others. The BCSS has been found to have good internal consistency (ranging from α = .78 to α = .88 across sub-scales in both clinical and non-clinical samples), good test–re-test reliability (ranging from *r* = .70 to *r =* .84) and good construct validity (Fowler *et al*., 2006). A 1-week time frame was used.

#### Social Comparison Scale (Allan and Gilbert, 1995; adapted by Freeman et al., 2014)

An adapted version of this self-report scale was used, which was developed specifically for this patient group (Freeman *et al*., 2014). It consists of 19 bipolar visual analogue scales, such as incompetent–competent, unlikeable–likeable, powerless–powerful, odd–normal, failure–success. Each item is scored on a scale of 0–100. For example, participants rate ‘in relation to others I have felt’: ‘inferior’ (scored as 0), ‘neither’ (scored as 50) or ‘superior’ (scored as 100). Total scores range from 0 to 1900. The adapted scale has good internal consistency (α = .93, .94 and .97 across three time points; Freeman *et al*., 2014). It uses a time frame of 1 week.

#### Rosenberg Self-Esteem Scale (Rosenberg, 1965)

This 10-item self-report questionnaire measures global self-esteem. Participants rate their level of agreement with each statement (e.g. ‘I feel that I am a person of worth, at least on an equal plane with others’) on a Likert scale of 1 (strongly agree) to 4 (strongly disagree). The higher the overall score, the lower the self-esteem. The Rosenberg Self-Esteem Scale has been shown to have good internal consistency for people with severe mental health problems (α = .88; Corrigan *et al*., 2011), and has been argued to have good construct validity (Rosenberg, 1965). It does not usually specify a time frame, but in this study a 1-week period was used.

### Intervention

The four-session compassionate imagery intervention was developed in collaboration with people with lived experience of psychosis (Oxford Cognitive Approaches to Psychosis Patient Advisory Group facilitated by the McPin Foundation). This included the focus of treatment, the exercises used, the materials, and the time frame of treatment delivery. The intervention was delivered 1:1 by a clinical psychologist in training (A.F.) under the supervision of a clinical psychologist (F.W.). It was delivered twice a week for 2 weeks, with each session typically lasting an hour.

The intervention used a series of adapted self-compassion imagery exercises (Gilbert, 2005; Gilbert, 2010a,b; Kolts, 2012; Lee and James, 2012; Neff, 2011; Welford, 2012). The adaptions included an emphasis on explaining the link between the threat system and persecutory beliefs, focusing on compassionate qualities that enabled participants to feel safer, and imagining their ‘compassionate coach’ providing support in social situations in which they would usually feel persecuted. The key approaches were:

- Psycho-education about anxiety, threat anticipation and responses;
- An introduction to mental imagery including a colour breathing exercise;
- Developing an image of the ‘compassionate coach’ (or ‘perfect nurturer’);
- Imagery exercises of the ‘compassionate coach’ in an anxiety-provoking social situation;
- Using the ‘compassionate coach’ in an everyday anxiety-provoking social situation;
- Creating a therapy blueprint.

The therapy manuals, used collaboratively in-session and given to participants as handouts, were developed as patient-facing documents. Participants received one handout per session, including a summary of the key techniques, space to take notes of in-session reflections, and reminders of between-session practice. In-session imagery exercises relating to the ‘compassionate coach’ became increasingly detailed and individualised. For example, in one session, an individualised list of anxiety-provoking social situations was developed, then a detailed plan on how to use the ‘compassionate coach’ in those situations was generated collaboratively. This formed the basis for the between-session practice to enter these situations with confidence. Audio recordings of the imagery scripts were provided on an mp3 player or CD to use between sessions. In sessions, the scripts were adapted to incorporate more detailed descriptions of each participant’s individual ‘compassionate coach’. At the end of therapy, participants were sent a summary letter and personalised recording of the imagery scripts. Participants were sent text message reminders of all appointments. Further detail on the content of each session is provided in the Supplementary material (Supplementary document 1).

#### Treatment adherence and competence

To assess fidelity, the content of each session was monitored using an adherence scale detailing the key techniques to be covered in each session. Sessions were audio recorded when permission was provided. To assess treatment quality, recordings of therapy sessions were rated on the Cognitive Therapy Scale-Revised (CTS-R; Blackburn *et al*., 2001) by F.W.

### Analysis

The primary focus of this study was the feasibility and acceptability of the intervention, as measured by descriptive statistics for recruitment rate, treatment uptake, and data completion, quantitative information from the feedback questionnaire and qualitative interviews. To provide a preliminary estimate of clinical efficacy, repeated measures ANOVAs were conducted on the clinical outcomes to analyse changes over time, with planned comparisons between the baseline and post-treatment assessment to analyse any treatment effects, and between the baseline and follow-up assessment to determine whether any effects remained at follow-up. Following recommendations that pilot studies should focus on confidence interval estimation (Lancaster *et al*., 2004), *p*-values are not reported. All statistical analyses were conducted using SPSS for Windows, version 24.0 (IBM Corporation, 2016).

#### Thematic analysis

Thematic analysis (Braun and Clarke, 2006) was used for organising, encoding and analysing patterns in the qualitative data. Initial codes and candidate themes were derived from the data by P.B.; F.W., who was independent of the therapy delivery and interviews, reviewed the analysis, performed credibility checks on the coding, and contributed to the identification of the final themes and thematic map. An example of an early codebook is provided in the Supplementary materials (Table S1 and supplementary document 2). Bracketing interviews and a reflexive log (see Supplementary material: supplementary document 3) were completed by P.B. and A.F.

## Results

### Basic demographic and clinical information

Five participants were female and seven were male, with a mean age of 42.0 years (*SD* = 13.1). Eleven participants identified as White British and one as Lebanese. Most participants were single (*n* = 9), with others married (*n* = 1), divorced (*n* = 1) or widowed (*n* = 1). Seven participants were living alone, one participant lived with a spouse and children, and four participants lived with their parents. Most participants were unemployed (*n =* 7), with one participant employed full-time, one participant employed part-time, one participant self-employed, one participant was a student, and one participant was a housewife/husband. Eleven participants were taking anti-psychotic medication. The participants’ primary diagnoses were schizophrenia (*n* = 9), first episode psychosis (*n* = 1), and psychosis not otherwise specified (*n* = 2). At baseline, the mean score on the GPTS Part B met the cut-off for persecutory delusions (mean = 41.57, *SD* = 16.41).

### Acceptability and feasibility

#### Recruitment rate

Seventeen people were referred to the study. Two people declined to meet for an eligibility screening and one person could not be contacted. Of the 14 patients who were screened, 12 agreed to participate (an 86% uptake rate).

#### Completion rate

The completion rate of all assessments and in-session measures was 100%. There were only six questions across three measures that were left blank by one participant, for which mean imputations were used.

#### Uptake of the intervention

There were no non-attendances or cancellations, yielding a 100% attendance rate. All participants completed all the planned content outlined in the therapy manuals, including all four imagery exercises. Participants were provided with a handout at the end of each session summarising the key content (for example, the imagery exercise covered in-session and a detailed plan on how to use this in daily life). Each of the four booklets provided space for participants to note their own reflections and learning. There was some flexibility in the order in which imagery exercises were used. The exercises in the final session were led by patient preference and included at least one of the imagery exercises covered in the previous sessions. Audio recordings of the sessions were rated as providing at least satisfactory cognitive therapy on the CTS-R (Blackburn *et al*., 2001) and adherent to the treatment techniques outlined in the therapy booklets.

#### Feedback on the intervention

At the post-treatment assessment, participants completed a feedback questionnaire rating their experience of therapy on a scale of 1 to 7, with 7 being the most positive rating. Feedback was positive, for example the mean rating was 6.58 (*SD* = 0.67) for both the overall treatment and likelihood of recommending the intervention to others (see Supplementary material: Table S2). Participants indicated that on average they practised the imagery exercises two to three times per week. There were high ratings for intervention understandability (mean = 6.58, *SD* = 0.67) and helpfulness (mean = 6.00, *SD* = 0.95).

#### Thematic analysis of qualitative interviews

All 12 participants agreed to take part in the qualitative interview. The semi-structured interviews lasted up to 40 min (mean duration = 18 min, SD = 10 min). Interviews took place either at the participant’s home or at their local clinic, with only the interviewer and participant present in each instance. The interviewer took field notes during and after each interview, and interviews were audio-recorded and transcribed verbatim. Three overall themes, each with sub-themes, were developed (see Supplementary material: Fig. S1). Examples of participant quotes supporting each theme and sub-theme can be seen in Table 1. A list of codes identified for each participant is provided in the Supplementary material (Table S1 and supplementary document 2).

**Table 1.** Illustrative participant quotes for each theme and sub-theme

**Theme 1: Effortful learning**
**Willingness to try** ‘I’ve thrown myself into it’ (participant 9) ‘I tried to build upon that diagram’ (participant 5) **Regular practice** ‘I did do it regularly’ (participant 10) ‘I have been using the techniques’ (participant 4)
**It can be difficult** ‘I found it difficult to believe in my compassionate coach … I had trouble visualising him’ (participant 5) ‘If things are happening quickly, it’s a bit difficult’ (participant 3) **Fears disrupting engagement** ‘When I was playing the CD, the neighbours, the nasty ones didn’t like it one bit… they were quite opposed to my playing the CD tape’ (participant 3) ‘I try and be careful not to play it too loud or whatever, because people will know what you’re doing and what’s going on’ (participant 4)
**Theme 2: Seeing change**
**Feeling calm** ‘I’ve definitely felt that I can make myself feel more relaxed’ (participant 1) ‘I see the coach as more of a humorous background that sort of deflects any anxiety I might be feeling’ (participant 10)
**Gaining clarity** ‘I’ve become more objective… I’m seeing things more rationally’ (participant 3) ‘I’ve been… more eloquent, speaking clearer’ (participant 4) ‘I’ve got control over my own brain’ (participant 6) **New perspectives** ‘[I’m] looking at things different and not getting so wound up’ (participant 9) ‘It makes me feel sort of almost quite realistic… even your worst fears aren’t that bad’ (participant 11) **Ability to cope** ‘I’m definitely learning different ways to manage better… I feel like I can manage [my voices]’ (participant 9) ‘I’m better able to cope with things when they happen’ (participant 3) ‘I felt stronger and more able to tackle problems’ (participant 11)
**Experiencing acceptance** - **Accepting others** ‘The compassion made me kinder towards people’ (participant 3) ‘[It’s] helped me engage more with people’ (participant 2) ‘It’s really nice… to accept someone being nice to you’ (participant 9) ‘I don’t feel quite so threatened as I did. I feel a little bit safer, I would say; maybe a little less vulnerable’ (participant 8) ‘I don’t need to worry about anybody else and what they’re thinking… I’m not as paranoid’ (participant 9) ‘It doesn’t change, really, what I think other people think of me’ (participant 1) ‘I still… fear someone’s going to shoot me’ (participant 12) - **Accepting self** ‘It just brings a lot of love to myself and actually to believe that has been really nice’ (participant 9) ‘Spending time alone isn’t really that much of a big deal… [it] helped me be more at ease with myself… I felt better in my skin when I was doing the [therapy]… I’ve been having more positive self-talk with myself’ (participant 2) ‘I feel like I can take part in life and things now, which I couldn’t really do before’ (participant 8) ‘Since I’ve started doing it [the study] I’ve felt quite confident… the confidence has stayed with me’ (participant 3)
**Theme 3: Taking it forward**
**Becoming ingrained** ‘I felt like it was strong and wise, I wasn’t just thinking, oh it’s a strong and wise globe, I actually felt that way’ (participant 11) ‘I don’t think, oh, I need to do that if that’s going to make me feel better, it’s just automatic’ (participant 9)
**Requires perseverance** ‘Definitely something I’m going to persevere with… the whole time I did appreciate it’s worth persevering with… you’ve got to persevere with it, definitely. I find that persevering has been worthwhile’ (participant 5) ‘I should try and use it more when I’m in difficult situations, I think, and I will’ (participant 4)

#### Theme 1: Effortful learning

The first theme reflected the process of engaging in and learning the therapeutic techniques. Participants described the difficulty of using the techniques, while also expressing a willingness to learn and to practise regularly: ‘*I’ve thrown myself in to it*’ (participant 9). For a number of participants, the main difficulty concerned visualisation [‘*I had trouble visualising him* [compassionate coach]’ (participant 3)] and one participant described finding it hard to focus during the longest exercise: ‘*the longer one; I feel like my concentration wasn’t as good, I wasn’t able to engage with it for the full length of time*’ (participant 5). For others, difficulty stemmed from their persecutory fears: ‘*When I was playing the CD, the neighbours, the nasty ones didn’t like it one bit … they were quite opposed to my playing the CD tape*’ (participant 3). Interestingly there was little comment on the pace or brevity of the intervention.

#### Theme 2: Seeing change

The second theme highlighted the changes that participants identified post-treatment. The first sub-theme ‘feeling calm’ was experienced by nearly all participants. Similarly, most participants described some sort of cognitive shift, for example gaining clarity [‘*I’m able to rationalise now*’ (participant 9)] or new perspectives [‘*It makes me feel… quite realistic… even your worst fears aren’t that bad*’ (participant 11)], or developing better coping mechanisms [‘*I’m better able to cope with things when they happen*’ (participant 3)].

Experiencing acceptance was the second sub-theme; this included acceptance of the self and acceptance of others. Self-acceptance was reported by many participants, depicted by increased self-compassion, confidence, and ease with self: ‘[the therapy] *helped me be more at ease with myself*’ (participant 2). Changes in confidence were attributed to the therapy work: ‘*Since I’ve started doing* [the therapy] *I’ve felt quite confident … the confidence has stayed with me*’ (participant 3). Importantly, changes in confidence also led to more engagement in everyday activities: ‘*I feel like I can take part in life and things now, which I couldn’t really do before*’ (participant 8), which was partially accounted for by a greater sense of safety: ‘*I don’t feel quite so threatened as I did. I feel a little bit safer, I would say, maybe a little less vulnerable*’ (participant 8). However, there were clear differences between participants in their experience of change in acceptance of others. For example, one participant reported: ‘*I don’t need to worry about anybody else and what they’re thinking… I’m not as paranoid*’ (participant 9), whereas another participant said: ‘*It doesn’t change, really, what I think other people think of me*’ (participant 1). Those who did report increased acceptance of others were more likely to have also described increased self-acceptance; whereas those who felt their worries about others were unchanged did not.

#### Theme 3: Taking it forward

The third theme focused on continuing to develop and use the key learning from therapy. Within this theme there were sub-themes on the techniques becoming ingrained and automatic [‘*I wasn’t just thinking, oh it’s a strong and wise globe, I actually felt that way*’ (participant 11); ‘*I don’t think Oh I need to do that if that’s going to make me feel better, it’s just automatic*’ (participant 9)], and another of the need, but also desire, to persevere with the strategies: ‘*Definitely something I’m going to persevere with… the whole time I did appreciate it’s worth persevering with… you’ve got to persevere with it, definitely. I find that persevering has been worthwhile*’ (participant 5).

#### Adverse events

There were no adverse events.

#### Preliminary clinical outcomes

##### Changes in self-compassion, negative self, and paranoia

Table 2 displays the means, standard deviations, change scores, confidence intervals for the change scores, and effect sizes for the clinical outcome measures at baseline (0 weeks), post-treatment (2 weeks) and follow-up (6 weeks). Post-treatment, there were large effect size improvements in self-compassion (change score –0.64, 95% CI –1.04, –0.24, *d* = –1.78), and medium effect size improvements in negative beliefs about the self (change score 2.42, 95% CI –0.37, 5.20, *d* = 0.51) and paranoia (change score 10.08, 95% CI 3.47, 16.69, *d* = 0.61). Improvements were maintained at follow-up.

**Table 2.** Means, standard deviations (*SD*), change scores, 95% confidence intervals (CI) for the change scores and estimated effect sizes (*d*) for pairwise comparisons on clinical outcome measures

Measure	Baseline *vs* post-treatment						Baseline *vs* follow-up					
	*n*	Mean (*SD*) at baseline	Mean (*SD*) at end of treatment	Change score	95% CI	*d*	*n*	Mean (*SD*) at baseline	Mean (*SD*) at follow-up	Change score	95% CI	*d*
Self-Compassion Scale	12	2.51 (0.36)	−3.15 (0.77)	−0.64	−1.04, –0.24	−1.78	12	2.51 (0.36)	2.91 (0.62)	−0.40	−0.71, –0.09	−1.10
BCSS negative-self	12	10.67 (4.77)	8.25 (5.10)	2.42	−0.37, 5.20	0.51	12	10.67 (4.77)	8.58 (5.32)	2.08	0.20, 3.97	0.44
GPTS Part B (paranoia)	12	45.17 (16.41)	35.08 (15.67)	10.08	3.47, 16.69	0.61	12	45.17 (16.41)	38.33 (19.48)	6.83	−1.72, 15.39	0.42
GPTS Total	12	88.50 (25.51)	67.75 (26.72)	20.75	9.12, 32.38	0.81	12	88.50 (25.51)	75.60 (35.79)	12.90	−1.22, 27.02	0.51
Social Comparison Scale	12	527.17 (307.56)	852.08 (500.37)	−324.92	−528.65, –121.19	−1.06	12	527.17 (307.56)	867.61 (495.91)	−340.44	−538.16, –142.72	−1.11
Rosenberg Self-Esteem Scale	12	28.17 (4.49)	24.58 (6.16)	3.58	1.51, 5.65	0.80	12	28.17 (4.49)	24.92 (5.93)	3.25	0.61, 5.89	0.72
BCSS positive-self	12	6.92 (4.44)	10.17 (5.64)	−3.25	−5.37, –1.13	−0.73	12	6.92 (4.44)	9.50 (5.92)	−2.58	−4.76, –0.41	−0.58
BCSS positive-others	12	9.25 (3.11)	10.83 (5.61)	−1.58	−4.23, 1.06	−0.51	12	9.25 (3.11)	10.33 (5.03)	−1.08	−3.90, 1.74	−0.35
BCSS negative-others	12	13.33 (3.52)	9.42 (5.43)	3.92	1.56, 6.27	1.11	12	13.33 (3.52)	8.75 (4.31)	4.58	2.01, 7.16	1.30

BCSS, Brief Core Schema Scale; GPTS, Green *et al*. Paranoid Thoughts Scale.

##### Changes in social comparison, self-esteem, positive self, and beliefs about others

Post-treatment, there were large effect size improvements in social comparison (change score –324.92, 95% CI –528.65, –121.19, *d* = –1.06) and self-esteem (change score 3.58, 95% CI 1.51, 5.65, *d* = 0.80). There were medium effect size improvements in positive beliefs about the self (change score –3.25, 95% CI –5.37, –1.13, *d* = –0.73) and others (change score –1.58, 95% CI –4.23, 1.06, *d* = –0.51). There were large effect size improvements in negative beliefs about others (change score 3.92, 95% CI 1.56, 6.27, *d* = 1.11). Gains were maintained at follow-up.

## Discussion

Paranoia builds upon the sense of vulnerability brought about by negative beliefs about the self. Hence improving views of the self is likely to reduce paranoia, as evidence from experimental studies and clinical intervention is beginning to demonstrate (Atherton *et al*., 2016; Freeman *et al*., 2014). To our knowledge, this was the first investigation of a repeated compassionate imagery intervention for patients with current persecutory delusions. The four-session compassionate imagery intervention was both feasible and acceptable. Patients described therapy as effortful yet rewarding, fuelling a commitment to continue implementing the techniques learned. The preliminary clinical effects were promising; there were medium to large effect size improvements on all clinical outcome measures: self-compassion improved, negative self-beliefs reduced, and paranoia came down. The effects were maintained at follow-up. Based on these preliminary findings the brief compassionate imagery intervention could be clinically beneficial and merits further testing.

The recruitment rate, the treatment uptake, and the rate of data completion were all very high, and participants gave positive feedback both in the questionnaire and the qualitative interview. This suggests that patients might be interested in receiving this treatment if it was offered in a larger trial. This is in line with recent survey data in which patients expressed the desire to address low self-confidence in therapy (Freeman *et al*., 2019). In terms of feasibility, future studies may need to establish clinicians’ willingness to refer and patients’ willingness to be randomised if the treatment was evaluated in a clinical trial.

In contrast to previous research (e.g. Martins *et al*., 2017; Martins *et al*., 2018), patients in this study did not report fear of compassion. Instead, the qualitative accounts highlight improvements in self-compassion and self-acceptance, which for many also extended to increased compassion for others. Within the preliminary clinical outcomes, there were medium to large effect sizes on all measures at end of treatment, showing an improvement in beliefs about the self and others, including improved self-compassion and self-esteem, and a reduction in paranoia. Consistent with our cognitive understanding of persecutory delusions (Freeman, 2016), the findings indicate that targeting the putative causal mechanism of negative self-beliefs leads to a reduction in paranoia.

This intervention focused on a single therapeutic technique: self-compassionate imagery. Yet CFT includes a range of techniques (see Gilbert, 2005; Gilbert, 2010) that may be effective in increasing a felt sense of safety and reducing threat beliefs. For example, in a robust non-clinical experimental test, loving kindness meditation, a compassionate imagery technique targeting compassion for others, increased compassion and reduced paranoia (Brown *et al*., 2020). Evaluations of CFT interventions using multiple techniques with patients with psychosis are currently underway (e.g. Heriot-Maitland *et al*., 2019). Other imagery techniques are also being adapted for people with first episode psychosis (Taylor *et al*., 2020).

The advantages of the brevity and intensity of this compassionate imagery intervention are the clarity in the treatment focus, and the opportunity for frequent practice within a short time frame to optimise learning, in line with studies which show that frequent rehearsal is important for the effectiveness of imagery exercises (Hackmann *et al*., 2011). As shown by the promising results on the clinical outcome measures, this leads to change in a short time frame for patients who have been struggling for a long time, thus potentially instilling hope.

There are clear limitations to this study. This was an uncontrolled feasibility study with a small sample size, a single therapist, and unblinded assessments. Therefore, the improvements made post-therapy cannot be attributed to the intervention with any certainty and all clinical outcomes need to be interpreted tentatively. A future study would need to be conducted with greater rigour, randomising patients to the intervention or a control arm, ensuring that assessors are blind to the treatment allocation, as well as being sufficiently powered. Mediation analyses would enable researchers to check that, as hypothesised, decreases in paranoia are explained by improvements in negative self-beliefs (Dunn *et al*., 2015).

The importance of the self-concept in the occurrence of paranoia has been demonstrated repeatedly; a clear direction for future clinical research is establishing evidence-based techniques to improve views of the self. Compassionate imagery may provide one avenue to achieve this. Robust clinical tests are now required.

## Data Availability

The data that support the findings of this study are available from the corresponding author, F.W., upon reasonable request.

## Supplementary material

For supplementary material accompanying this paper visit https://doi.org/10.1017/S1352465821000229. click here to view supplementary material

## References

[ref1] Allan, S. , & Gilbert, P. (1995). A social comparison scale: psychometric properties and relationship to psychopathology. Personality and Individual Differences, 19, 293–299. 10.1016/0191-8869(95)00086-L

[ref2] Ascone, L. , Sundag, J. , Schlier, B. , & Lincoln, T. M. (2017). Feasibility and effects of a brief compassion-focused imagery intervention in psychotic patients with paranoid ideation: a randomized experimental pilot study. Clinical Psychology and Psychotherapy, 24, 348–358. 10.1002/cpp.2003 26888312

[ref3] Atherton, S. , Antley, A. , Evans, N. , Cernis, E. , Lister, R. , Dunn, G. , … & Freeman, D. (2016). Self-confidence and paranoia: an experimental study using an immersive virtual reality social situation. Behavioural and Cognitive Psychotherapy, 44, 56–64. 10.1017/S1352465814000496 25384608

[ref4] Blackburn, I. M. , James, I. A. , Milne, D. L. , Baker, C. , Standart, S. , Garland, A. , & Reichelt, F. K. (2001). The revised cognitive therapy scale (CTS-R): psychometric properties. Behavioural and Cognitive Psychotherapy, 29, 431. doi: 10.1017/S1352465801004040

[ref5] Braun, V. , & Clarke, V. (2006). Using thematic analysis in psychology. Qualitative Research in Psychology, 3, 77–101. 10.1191/1478088706qp063oa

[ref6] Brown, P. , Waite, F. , Rovira, A. , Nickless, A. , & Freeman, D. (2020). Virtual reality clinical-experimental tests of compassion treatment techniques to reduce paranoia. Scientific Reports, 10, 8547. 10.1038/s41598-020-64957-7 32444619 PMC7244556

[ref7] Collett, N. , Pugh, K. , Waite, F. , & Freeman, D. (2016). Negative cognitions about the self in patients with persecutory delusions: an empirical study of self-compassion, self-stigma, schematic beliefs, self-esteem, fear of madness, and suicidal ideation. Psychiatry Research, 239, 79–84. 10.1016/j.psychres.2016.02.043 27137965

[ref8] Corrigan, P. W. , Rafacz, J. , & Rüsch, N. (2011). Examining a progressive model of self-stigma and its impact on people with serious mental illness. Psychiatry Research, 189, 339–343. 10.1016/j.psychres.2011.05.024 21715017 PMC3185170

[ref9] Dunn, G. , Emsley, R. , Liu, H. , Landau, S. , Green, J. , White, I. , & Pickles, A. (2015). Evaluation and validation of social and psychological markers in randomised trials of complex interventions in mental health: a methodological research programme. *Health Technology Assessment*, *19*. 10.3310/hta19930 PMC4781463 26560448

[ref10] Fowler, D. , Freeman, D. , Smith, B. , Kuipers, E. , Bebbington, P. , Bashforth, H. , … & Garety, P. (2006). The Brief Core Schema Scales (BCSS): psychometric properties and associations with paranoia and grandiosity in non-clinical and psychosis samples. *Psychological Medicine*. 10.1017/S0033291706007355 16563204

[ref11] Freeman, D. (2007). Suspicious minds: the psychology of persecutory delusions. Clinical Psychology Review, 27, 425–457. 10.1016/j.cpr.2006.10.004 17258852

[ref12] Freeman, D. (2016). Persecutory delusions: a cognitive perspective on understanding and treatment. The Lancet Psychiatry, 3, 685–692. 10.1016/S2215-0366(16)00066-3 27371990

[ref13] Freeman, D. , Loe, B. , Kingdon, D. , Startup, H. , Molodynski, A. , Rosebrock, L. , Brown, P. , Sheaves, B. , Waite., F. , & Bird, J. (2021). The revised Green et al. Paranoid Thoughts Scale (R-GPTS): psychometric properties, severity ranges, and clinical cut-offs. Psychological Medicine, 51, 244–253. doi: 10.1017/S0033291719003155 31744588 PMC7893506

[ref14] Freeman, D. , Pugh, K. , Dunn, G. , Evans, N. , Sheaves, B. , Waite, F. , … & Fowler, D. (2014). An early Phase II randomised controlled trial testing the effect on persecutory delusions of using CBT to reduce negative cognitions about the self: the potential benefits of enhancing self confidence. Schizophrenia Research, 160, 186–192. 10.1016/j.schres.2014.10.038 25468186 PMC4266450

[ref15] Freeman, D. , Taylor, K. M. , Molodynski, A. , & Waite, F. (2019). Treatable clinical intervention targets for patients with schizophrenia. Schizophrenia Research, 211, 44–50. 10.1016/j.schres.2019.07.016 31326234

[ref16] Garety, P. A. , Ward, T. , Freeman, D. , Fowler, D. , Emsley, R. , Dunn, G. , … & Hardy, A. (2017). SlowMo, a digital therapy targeting reasoning in paranoia, versus treatment as usual in the treatment of people who fear harm from others: study protocol for a randomised controlled trial. Trials, 18, 1–13. 10.1186/s13063-017-2242-7 29096681 PMC5667466

[ref17] Garety, P. , & Freeman, D. (1999). Cognitive approaches to delusions: a critical review of theories and evidence. British Journal of Clinical Psychology, 38, 113–154. 10.1348/014466599162700 10389596

[ref18] Gilbert, P. (2005). Compassion: Conceptualisations, Research and Use in Psychotherapy. London, UK: Routledge.

[ref19] Gilbert, P. (2009). Introducing compassion-focused therapy. Advances in Psychiatric Treatment, 15, 199–208. 10.1192/apt.bp.107.005264

[ref20] Gilbert, P. (2010a). Compassion Focused Therapy: Distinctive Features. Hove: Routledge.

[ref21] Gilbert, P. (2010b). The Compassionate Mind: A New Approach to Life’s Challenges. Oaklands, CA, USA: New Harbinger Publications.

[ref22] Green, C. E. L. , Freeman, D. , Kuipers, E. , Bebbington, P. , Fowler, D. , Dunn, G. , & Garety, P. A. (2008). Measuring ideas of persecution and social reference: the Green et al. Paranoid Thought Scales (GPTS). Psychological Medicine, 38, 101–111. 10.1017/S0033291707001638 17903336

[ref23] Hackmann, A. , Bennett-Levy, J. , & Holmes, E. A. (2011). Oxford Guide to Imagery in Cognitive Therapy. Oxford, UK: Oxford University Press. 10.1093/med:psych/9780199234028.001.0001

[ref39] Heriot-Maitland, C. , McCarthy-Jones, S. , Longden, E. , & Gilbert, P. (2019). Compassion focused approaches to working with distressing voices. Frontiers in Psychology, 10, 152. 30774614 10.3389/fpsyg.2019.00152 PMC6367219

[ref24] Kesting, M.-L. , & Lincoln, T. M. (2013). The relevance of self-esteem and self-schemas to persecutory delusions: a systematic review. Comprehensive Psychiatry, 54, 766–789. 10.1016/j.comppsych.2013.03.002 23684547

[ref25] Kolts, R. (2012). The Compassionate Mind Approach to Managing your Anger. London, UK: Hachette.

[ref26] Lancaster, G. A. , Dodd, S. , & Williamson, P. R. (2004). Design and analysis of pilot studies:recommendations for good practice. Journal of Evaluation in Clinical Practice, 10, 307–312. 10.1111/j.2002.384.doc.x 15189396

[ref27] Lee, D. , & James, S. (2012). The Compassionate Mind Approach to Recovering from Trauma. London, UK: Robinson.

[ref28] Lincoln, T. M. , Hohenhaus, F. , & Hartmann, M. (2013). Can paranoid thoughts be reduced by targeting negative emotions and self-esteem? An experimental investigation of a brief compassion-focused intervention. Cognitive Therapy and Research, 37, 390–402. 10.1007/s10608-012-9470-7

[ref29] Martins, M. J. , Barreto Carvalho, C. , Macedo, A. , Pereira, A. T. , Braehler, C. , Gumley, A. , & Castilho, P. (2018). Recovery through affiliation: a COMPassionate Approach to Schizophrenia and Schizoaffective disorder (COMPASS). Journal of Contextual Behavioral Science, 9, 97–102. 10.1016/j.jcbs.2018.07.009

[ref30] Martins, M. J. , Castilho, P. , Carvalho, C. B. , Pereira, A. T. , Carvalho, D. , Bajouco, M. , … & Macedo, A. (2017). Pathways from paranoid conviction to distress: exploring the mediator role of Fears of Compassion in a sample of people with psychosis. Psychosis, 9, 330–337. 10.1080/17522439.2017.1349830

[ref31] Neff, K. (2003a). Self-compassion: an alternative conceptualization of a healthy attitude toward oneself. Self and Identity, 2, 85–101. 10.1080/15298860309032

[ref32] Neff, K. (2003b). The development and validation of a scale to measure self-compassion. Self and Identity, 2, 223–250. 10.1080/15298860309027

[ref33] Neff, K. (2011). Self-Compassion. London, UK: Hachette.

[ref34] Rosenberg, M. (1965). Rosenberg Self-Esteem Scale (RSE). Measures Package, 61, 52. 10.1037/t01038-000

[ref35] Taylor, C. D. J. , Bee, P. E. , Kelly, J. , Emsley, R. , & Haddock, G. (2020). IMAgery focused psychological therapy for persecutory delusions in PSychosis (iMAPS): a multiple baseline experimental case series. *Behavioural and Cognitive Psychotherapy.* 10.1017/S1352465820000168 32264985

[ref36] Tiernan, B. , Tracey, R. , & Shannon, C. (2014). Paranoia and self-concepts in psychosis: a systematic review of the literature. Psychiatry Research, 216, 303–313. 10.1016/j.psychres.2014.02.003 24630916

[ref37] Tong, A. , Sainsbury, P. , & Craig, J. (2007). Consolidated criteria for reporting qualitative research (COREQ): a 32-item checklist for interviews and focus groups. International Journal for Quality in Health Care, 19, 349–357. 17872937 10.1093/intqhc/mzm042

[ref38] Welford, M. (2012). The Compassionate Mind Approach to Building Self-Confidence. London, UK: Hachette.

