# Supplementary material for: A compassionate imagery intervention for patients with persecutory delusions
Source: Behav Cogn Psychother. 2021 Jun 3;50(1):15–27. doi: 10.1017/S1352465821000229 (PMC9019554; doi:10.1017/S1352465821000229)
Supplement: Supplementary file 1 [file S1352465821000229sup001.zip › S1352465821000229supp001.docx]

Supplementary table 1: Illustrative individual codes from thematic analysis

| **Participant number** | **Illustrative codes identified from transcript** |
| --- | --- |
| 1 | Trying to practise, find it relaxing, loneliness, lack of change, trying out activities, self-care, feeling positive, hope, needing help, other people make it hard, importance of companionship, difficulty clarifying feelings |
| 2 | Feeling positive, engaging with others, doing activities, seeing improvement, feeling at ease with self, positive self-talk, lack of change re feelings about others, find it relaxing, find it helpful, have been practising |
| 3 | Improved confidence and happiness, others make it hard, difficulty visualising, hard to believe in the coach, memories brought up, feeling happy, feeling kinder towards others, trying to practise, coach became ingrained, coping better |
| 4 | Doing activities, improvement in confidence, practising, gaining clarity, being kind to self, this was needed, this has helped, others make it hard, feeling positive, finding it uplifted, some lack of change, |
| 5 | Trying to practise, helps me be strong, reduces anxiety, I can focus better, difficulty practising when down, understanding the science behind it, practising in difficult situations, difficulty visualising, perseverance, takes time, it’s worthwhile |
| 6 | Lack of clarity, confusion, low confidence, others are nasty, compassion gave me increased control over my brain, increased self-kindness, image of coach gave me companionship, will continue using, find it helpful, find it relaxing |
| 7 | Lack of change in self-compassion, find it relaxing, helps my confidence, have been practising, will use in the future |
| 8 | Improved confidence, easier on self, less self-critical, worries about others still, feel safer, less threatened and less vulnerable, have hope, been practising, helped at subconscious level, found helpful, good to tailor exercises to individuals |
| 9 | Feeling settled, coping better, improved confidence, able to rationalise better, found it relaxing, greater self-acceptance, less paranoid, improvement in voices, less worries, more self-care, seeing things differently, it was easy/automatic, will use in future, therapy gave me time to think, questionnaires increased my self-awareness, enjoyed it, wanted it to work |
| 10 | Regularly practising, improvement in voices, less worried about others, more positive, more focussed, feeling happier, trying out activities, feeling less anxious, found it helpful |
| 11 | Improved confidence, feeling calm, exercise became ingrained, feel stronger, enjoyed it, it’s given me perspective, will keep using |
| 12 | Have been using it, it’s helpful, still feel persecuted by others, feeling more positive, found it relaxing, felt safe during therapy, it was good therapy, feeling more self-compassionate |
