# Supplementary material for: A compassionate imagery intervention for patients with persecutory delusions
Source: Behav Cogn Psychother. 2021 Jun 3;50(1):15–27. doi: 10.1017/S1352465821000229 (PMC9019554; doi:10.1017/S1352465821000229)
Supplement: Supplementary file 1 [file S1352465821000229sup001.zip › S1352465821000229supp002.docx]

**Supplementary material: Thematic analysis –**

**initial candidate themes and illustrative quotes**

**Feelings about Others**

- lack of change (in worries/fears about others)

It doesn't change, really, what I think other people think of me. (ppt 1)

I still…fear someone’s going to shoot me. (ppt 12)

- improvement (less worried about others)

I don't feel quite so threatened as I did. I feel a little bit safer, I would say; maybe a little less vulnerable. (ppt 8)

I don’t need to worry about anybody else and what they’re thinking…I’m not as paranoid. (ppt 9)

- other people as a barrier to therapy

When I was playing the CD, the neighbours, the nasty one didn’t like it one bit…they were quite opposed to my playing the CD tape. (ppt 3)

I try and be careful not to play it too loud or whatever, because people will know what you’re doing and what’s going on. (ppt 4)

- acceptance of others

The compassion made me kinder towards people. (ppt 3)

Helped me engage more with people. (ppt 2)

It’s really nice…to accept someone being nice to you. (ppt 9)

**Value of Therapy**

- self-acceptance

It brings a lot of love to myself. I’m accepting myself a lot more. (ppt 9)

Spending time alone isn’t really that much of a big deal….helped me be more at ease with myself….i felt better in my skin when I was doing the work [therapy]. (ppt 2)

- relaxation

[I’m] relaxed in my mind at the minute, is the word, which is nice…I’m more relaxed about things. (ppt 9)

I’ve definitely felt that I can make myself feel more relaxed. (ppt 1)

- clarity/rationalisation

[I’m] able to rationalise. (ppt 9)

I’ve become more objective…I’m seeing things more rationally. (ppt 3)

I’ve been…more eloquent, speaking clearer. (ppt 4)

- perspective

[I’m] looking at things different and not getting so wound up. (ppt 9)

It kind of makes you view things differently…but in a much better light. (ppt 9)

It makes me feel sort of almost quite realistic…even your worst fears aren’t that bad. (Ppt 11)

- ability to cope

I’m definitely learning different ways to manage better. (ppt 9)

I feel like I can manage [my voices]. (ppt 9)

I’m better able to cope with things when they happen. (ppt 3)

I felt stronger and more able to tackle problems. (ppt 11)

- improvements (confidence, compassion, anxiety)

I feel like I can take part in life and things now, which I couldn’t really do before. (ppt 8)

Since I’ve started doing it [the study] I’ve felt quite confident…the confidence has stayed with me. (ppt 3)

I’m a bit more compassionate towards myself now. (ppt 4)

I’ve been having more positive self-talk with myself. (ppt 2)

It just brings a lot of love to myself and actually to believe that has been really nice. (ppt 9)

I’ve found things like that are a lot easier to do now, because I’m not as anxious about things. (ppt 9)

I see the coach as more of a humorous background that sort of deflects any anxiety I might be feelings. (ppt 10)

I actually feel happy, which is quite nice, really. (ppt 3)

**Implementing Learning**

- - Trying to practise

I still try to practise it…trying to relate to her strength and wisdom. (ppt 5)

I have been using the techniques. (ppt 4)

I did do it regularly. (ppt 10)

- - Perseverance/ future use

Definitely something I’m going to persevere with…the whole time I did appreciate it’s worth persevering with…you’ve got to persevere with it, definitely. I find that persevering has been worth while. (ppt 5)

I know that I can use it in the future, I can use it when I need to. (ppt 9)

I should try and use it more when I’m in difficult situations, I think, and I will. (ppt 4)

- - willingness

I’ve thrown myself into it. (ppt 9)

I tried to build upon that diagram. (ppt 5)

- - difficulty

I found it difficult to believe in my compassionate coach…I had trouble visualising him. (ppt 3)

I did find that a bit harder to physically imagine. (ppt 5)

If things are happening quickly, it’s a bit difficult. (ppt 3)

- - Techniques being ingrained

I felt like it was strong and wise, I wasn’t just thinking, oh it’s a strong and wide globe, I actually felt that way. (ppt 11)
I don’t think, oh, I need to do that if that’s going to make me feel better, It’s just automatic. (ppt 9)
