# Supplementary material for: A compassionate imagery intervention for patients with persecutory delusions
Source: Behav Cogn Psychother. 2021 Jun 3;50(1):15–27. doi: 10.1017/S1352465821000229 (PMC9019554; doi:10.1017/S1352465821000229)
Supplement: Supplementary file 1 [file S1352465821000229sup001.zip › S1352465821000229supp003.docx]

Supplementary table 2*. Responses to the feedback questionnaire*

| Question | *N* | Mean | *SD* |
| --- | --- | --- | --- |
| Did the imagery exercises make sense? | 12 | 6.58 | 0.67 |
| How helpful were the imagery exercises in helping you feel safer? | 12 | 6.00 | 0.95 |
| How helpful were the imagery exercises in helping you feel more understanding of yourself? | 12 | 5.67 | 0.98 |
| How manageable was it to practise imagery exercises between each session? | 12 | 5.67 | 1.15 |
| Will you continue using these techniques? | 12 | 6.33 | 0.65 |
| How would you rate the treatment overall? | 12 | 6.58 | 0.67 |
| If this intervention were to be offered again, would you recommend it to others? | 12 | 6.58 | 0.67 |
