# Supplementary material for: A compassionate imagery intervention for patients with persecutory delusions
Source: Behav Cogn Psychother. 2021 Jun 3;50(1):15–27. doi: 10.1017/S1352465821000229 (PMC9019554; doi:10.1017/S1352465821000229)
Supplement: Supplementary file 1 [file S1352465821000229sup001.zip › S1352465821000229supp004.docx]

**Supplementary material: Thematic analysis –**

**Position statement and excerpt from reflexive log**

**Position statement – domain 1 of COREQ**

The interviewer, XX, was a female doctoral student working in the team. XX underwent training in qualitative interviewing and analysis methods by her supervisor XX in preparation for this work. Prior to data collection XX and XX conducted a bracketing interview in order to identify their expectations, assumptions, and potential biases. It should be acknowledged in particular that XX has also conducted research into compassion therapies as part of her doctoral research, and thus her existing knowledge and experience in this area may have guided her during the interviews and thematic analysis. For example, there may have been a greater expectation that the therapy would bring about positive change, given this was the outcome of her own research into compassion interventions for psychosis. However, to suspend this potential bias, XX ensured to stay close to the interview scheduled designed by XX, and refrained from asking any potentially leading questions. There were no prior relationships between XX and the participants. Participants were aware that XX was a colleague of XX and aware that the research would contribute towards an academic qualification for the first author.

**Excerpt from reflexive log**

XX maintained a log of field and reflexive notes. For example, with several participants she noted that during the interview they showed visible positive affect when talking about their compassionate coach, which suggested to her that the coach may have become very well ingrained for those participants and that they had experienced positive change. This helped contribute to the development of the ‘seeing change’ theme and the ‘becoming ingrained’ subtheme. However, with two others she noted that the emotional tone of the interview overall appeared more neutral and sometimes negative, which reinforced the importance of including a subtheme around lack of change, particularly with regards to feelings about other people.
