# Supplementary material for: A compassionate imagery intervention for patients with persecutory delusions
Source: Behav Cogn Psychother. 2021 Jun 3;50(1):15–27. doi: 10.1017/S1352465821000229 (PMC9019554; doi:10.1017/S1352465821000229)
Supplement: Supplementary file 1 [file S1352465821000229sup001.zip › S1352465821000229supp005.docx]

**Supplementary material: Overview of therapy**

The therapy manual for session 1 covered an explanation of the compassion model; a review of how active each of the three emotion regulation systems (‘circles’) in the compassion model currently was for each participant and how they would like this to be in the future; an explanation of imagery; and two imagery exercises – a colour breathing exercise and an introduction to the ‘compassionate coach’.

The therapy manual for session 2 contained a summary and space to reflect on the previous session; an individualised list of the qualities of compassion; and a ‘compassionate coach’ imagery exercise in which the qualities of compassion chosen by the individual participant were incorporated. Moreover, an individualised list of anxiety-provoking social situations was developed so as to practice imagining these situations with the ‘compassionate coach’ there to support them.

Therapy manual 3 built on this: participants practiced imagining those social situations while being supported by their ‘compassionate coach’ . Subsequently, a detailed plan on how to use the compassionate coach in those situations in real life was generated collaboratively. This formed the basis for the between-session practice to enter these situations with confidence.

Therapy manual 4 comprised a recap of the key techniques; a review of how active each of the three emotion regulation systems was for each participant now and how this compared to the start of therapy; and a detailed, individualised therapy blueprint. In addition, session 4 included the repetition of an imagery exercise of their choice (colour breathing, meeting the ‘compassionate coach’, or imagining the ‘compassionate coach’ supporting them through a difficult social situation).
