# Supplementary material for: A compassionate imagery intervention for patients with persecutory delusions
Source: Behav Cogn Psychother. 2021 Jun 3;50(1):15–27. doi: 10.1017/S1352465821000229 (PMC9019554; doi:10.1017/S1352465821000229)
Supplement: Supplementary file 1 [file S1352465821000229sup001.zip › S1352465821000229supp006.docx]

Supplementary measure 1*.* *Interview schedule.*

**An exploration of individuals’ subjective experiences of**

**a brief self-compassion intervention to tackle fears about other people**

**Main questions**

*(Prompts are in italics)*

1. Can you tell me a bit about how things are for you at the moment?

- *What have you been doing?*
- *How have you been feeling?*
- *How would you describe your confidence?*

1. Have there been any changes?

- *In your self-compassion? Confidence? Worries or fears about other people? Other areas?*
- *Has anyone else (friends or family) noticed a change?*

1. What was it like taking part for you?

- *Did you find anything more or less helpful?*
- *How did you find the therapy? How did you find the assessments?*
- *How did you find the practices between sessions (/homework)? Have you felt able to practice the imagery exercises? How did you find the handouts and audio recordings?*

1. Is there anything that we haven’t asked that you feel may have been important to your experience?

- *Any suggestions for the future? Anything else you would like to add?*
