# Supplementary material for: A compassionate imagery intervention for patients with persecutory delusions
Source: Behav Cogn Psychother. 2021 Jun 3;50(1):15–27. doi: 10.1017/S1352465821000229 (PMC9019554; doi:10.1017/S1352465821000229)
Supplement: Supplementary file 1 [file S1352465821000229sup001.zip › S1352465821000229supp007.docx]

*Supplementary figure 1*. Map of Thematic Analysis
